# DICER1 Syndrome With Embryonal Rhabdomyosarcoma of the Uterine Cervix and Retroperitoneal Metastasis: A Case Report and Literature Review

**DOI:** 10.1155/crog/9718758

**Published:** 2026-03-11

**Authors:** Xiao-qiang Wei, Qiu-yang Wang, Tian Li, You-bin Hu

**Affiliations:** ^1^ Department of Gynecology, Qingdao Women and Children′s Hospital, Women and Children′s Hospital of Qingdao University, Qingdao, Shandong, China; ^2^ Department of Obstetrics, Qingdao Chengyang People′s Hospital, Qingdao, Shandong, China

**Keywords:** case report, cervical embryonal rhabdomyosarcoma, DICER1 syndrome, prognosis

## Abstract

**Background:**

DICER1 syndrome is a rare autosomal dominant genetic disorder and presents a variety of manifestations.

**Case:**

A 15‐year‐old adolescent presented cervical embryonal rhabdomyosarcoma, retroperitoneal tumor and multinodular goiter. Genetic analysis demonstrated a mutation in Exon 25 of DICER1 gene, a mutation in Intron 19 of NF1 gene, and a mutation in Exon 7 of TP53 gene. The patient received surgical treatment and six courses of combination chemotherapy. After 7 months of initial diagnosis, the patient occurred a pleural and mediastinal metastasis and eventually died of respiratory failure.

**Conclusion:**

Multiple gene mutations, in addition to DICER1 gene mutation, may influence the behavior and prognosis of DICER1 syndrome. We detail the necessity of instituting personalized, multidisciplinary monitoring plans, including regular clinical evaluations and targeted imaging of high‐risk organs, to enable early detection and intervention.

## 1. Introduction

DICER1 syndrome (OMIM#601200) is a rare autosomal dominant predisposition syndrome caused by a heterozygous DICER1 germline mutation. In 1996, Priest et al. first linked pleuropulmonary blastoma (PPB) to an increased risk of various cancers [1]. In 2009, Hill et al. detected germline DICER1 mutations in familial PPB [2]. With subsequent studies of clinical cases, germline and somatic DICER1 mutations have been identified in conditions such as cystic nephroma, ovarian Sertoli‐Leydig cell tumor, pineoblastoma, hyperplastic proliferations like multinodular goiter or thyroid carcinoma, and embryonal rhabdomyosarcomas (RMS) of the female genital tract and other sites [3, 4].

RMS is a rare and highly aggressive soft tissue sarcoma primarily affecting children and young adults. Due to its histological subtypes, RMS is categorized as: embryonal, alveolar, pleomorphic, and spindle cell/sclerosing [5]. The most common site for embryonal rhabdomyosarcoma (ERMS) in the female genital tract is the vagina, with typical diagnosis occurring around the age of two. Cervical ERMS is rare and typically occurs in adolescents and young adults [6]. We present the case of a 15‐year‐old adolescent with cervical ERMS, retroperitoneal metastasis, and multinodular goiter. Molecular testing revealed a somatic DICER1 mutation. This manuscript describes the clinical presentation, imaging findings, pathological features, and therapeutic management of this case, also provides a review of the current literature on this topic.

## 2. Case Report

In February 2023, a 15‐year‐old virgin was admitted to the hospital with complaints of irregular vaginal bleeding and a vaginal mass for 3 months (Figure S1a). She did not report any other discomfort. Weight loss was also not reported. Exceptional medical or surgical history and the history for a family member with malignancy were not reported. A pelvic MRI scan revealed an exophytic mass (59 × 56 × 39 mm) (Figure S1c) located at the posterior wall of the cervical os, filling the vagina. Meanwhile, a solid mass (43 × 42 × 44 mm) was found in the right adnexal region of the pelvic cavity (Figure S1f), with no evidence of enlarged lymph nodes. Ultrasonography of the thyroid gland demonstrated multinodular goiter. The results of serum cancer antigen 125 (CA 125), alpha‐fetoprotein (AFP), cancer antigen 19‐9 (CA199), and human epididymis protein 4 (HE4) were all negative. Subsequently, hysteroscopy revealed a botryoid, fragile mass with dendritic vessels (Figure S1b). Biopsy confirmed botryoid ERMS.

Therefore, we performed a wedge resection of the cervical mass, laparoscopic retroperitoneal tumor resection, and right pelvic lymph node dissection (Figure S1d,e). Final pathology confirmed ERMS in both the cervical and retroperitoneal tumors (Figure 1a), with no lymph node metastasis. Immunohistochemical staining was positive for smooth muscle actin (SMA) (Figure 1d) and vimentin; partly positive for MyoD1 and myogenin (Figure 1b,c); but negative for CKpan, CD34, and CgA. The Ki‐67 proliferative index was approximately 60% (Figure 1e).

Figure 1Histopathology and immunohistochemistry (IHC) of the cERMS (×100). Densely packed and confluent aggregates of primitive rhabdomyoblast cells (a). Immunohistochemical staining was partly positive for MyoD1 (b), myogenin (c), and positive for SMA (d). The Ki67 proliferative index was about 60% (e).

**Figure:**
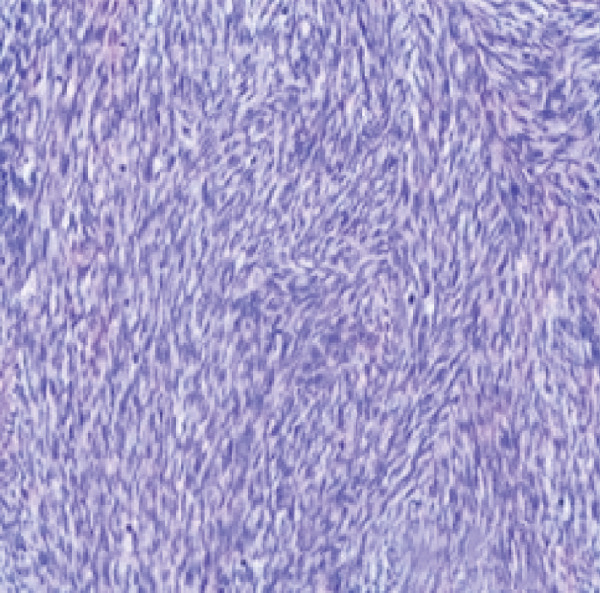
(a)

**Figure:**
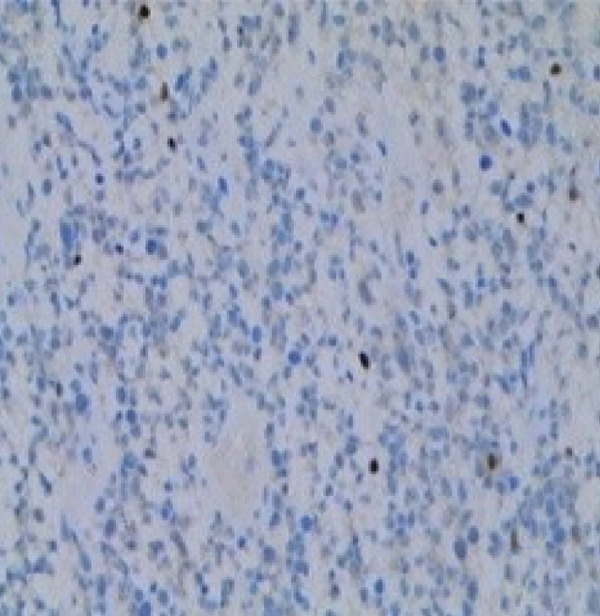
(b)

**Figure:**
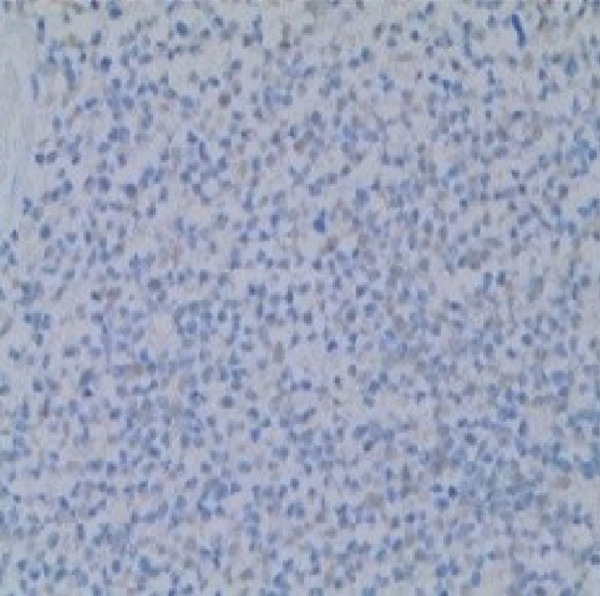
(c)

**Figure:**
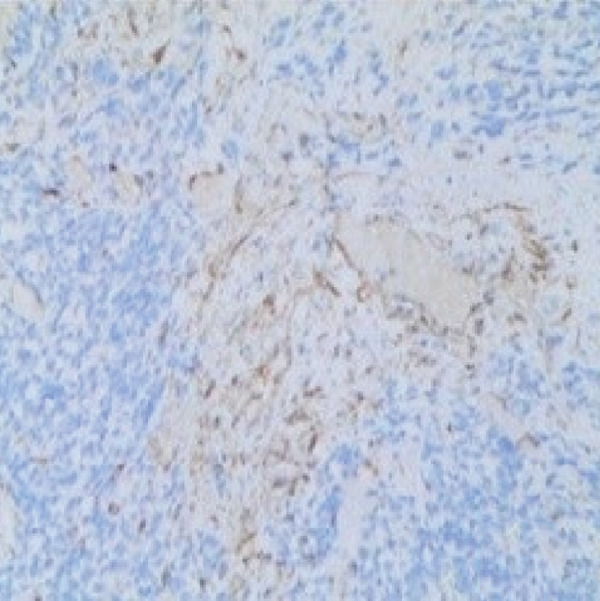
(d)

**Figure:**
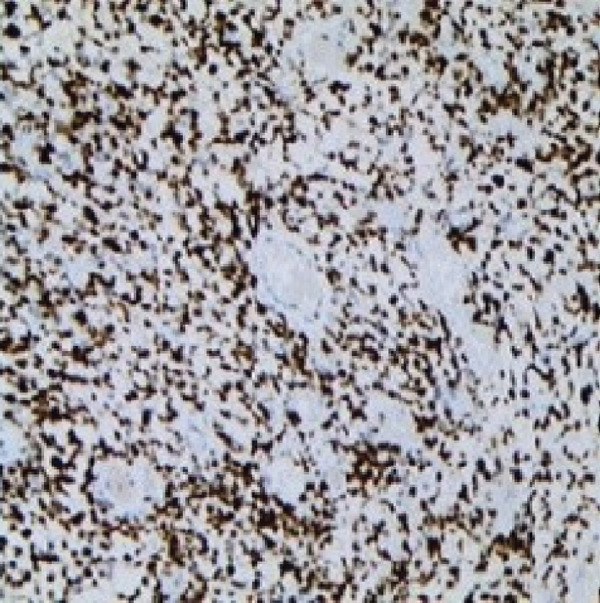
(e)

**Figure 2:**
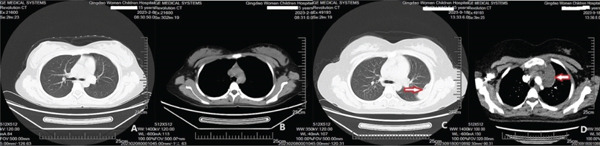
Chest CT in September revealed new pleural and mediastinal masses (C‐D) that were not present on imaging seven months prior (A‐B).

Molecular genetic analysis of the patient revealed a mutation (c.5439G > C, E1813D) in Exon 25 of the DICER1 gene, a mutation (c.2325+2T > A) in Intron 19 of the NF1 gene, and a mutation (c.710_718del) in Exon 7 of the TP53 gene. Based on the patient′s all medical information, the postoperative diagnosis is considered DICER1 syndrome with cervical ERMS and retroperitoneal metastasis. The patient was advised to undergo six courses of combination chemotherapy (ifosfamide, carboplatin, and etoposide). The patient experienced nausea, vomiting, and myelosuppression during chemotherapy but was able to tolerate and complete all scheduled combination chemotherapy. In September, chest CT scan revealed new left pleural and mediastinal masses (Figure 2c,d) that were not present on imaging 7 months prior (Figure 2a,b). A biopsy confirmed RMS (Figure 3a). Immunohistochemical staining showed partly positive for MyoD1, myogenin (Figure 3b,c). Given the patient′s history and the biopsy results, we believe this to be metastasis to the pleura and mediastinum from DICER1 syndrome. The patient′s condition was assessed as severe because metastases to the mediastinum and left chest wall occurred shortly after chemotherapy ended. Due to financial and personal reasons, the patient did not undergo further treatment and succumbed to respiratory failure in May 2024.

Figure 3Histopathology and immunohistochemistry (IHC) of the pleural and mediastinal tumor (×100). Densely packed and confluent aggregates of primitive rhabdomyoblast cells (a). Immunohistochemical staining were partly positive for MyoD1(b), myogenin (c).

**Figure:**
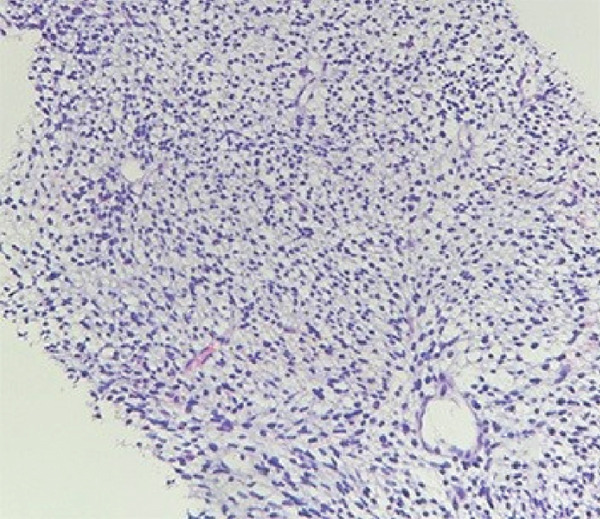
(a)

**Figure:**
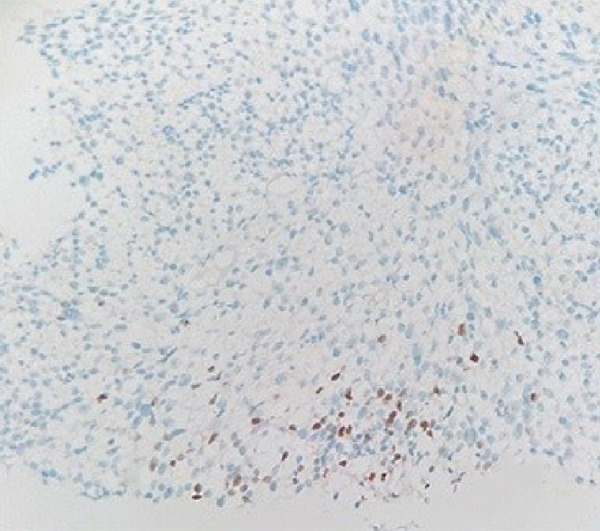
(b)

**Figure:**
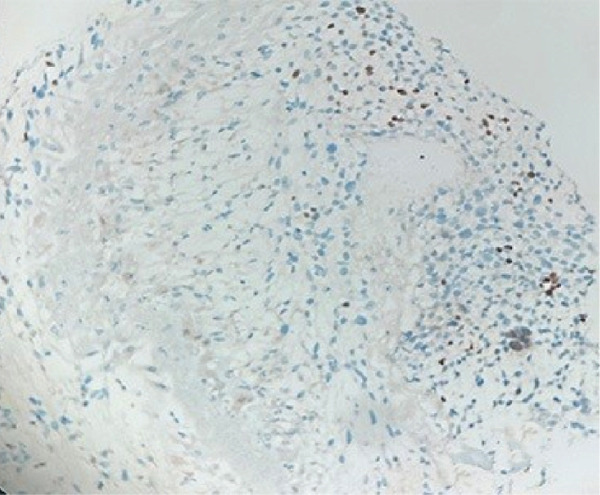
(c)

## 3. Discussion

The DICER1 gene is located on Chromosome 14q32.13 and encodes the DICER protein, an RNase III enzyme that processes pre‐miRNAs and double‐stranded RNA into functional miRNAs and small interfering RNAs (siRNAs) [7]. DICER1‐associated tumors often have germline loss‐of‐function mutations in one allele and somatic missense mutations in the other allele. Somatic mutations are concentrated in five hotspot codons located within Exons 24 and 25 (i.e., E1705, D1709, G1809, D1810, and E1813) [8]. The mutations affect conserved residues in the RNase IIIb domain and create a bias in miRNA production and ultimately drive tumorigenic potential in restricted tissue types [9].

Yoon et al. [10] believe that the cervical ERMS arose within conventional benign endocervical polyps. They also suggest that the presence of abnormal cellular and/or atypical stromal elements, coupled with positive immunohistochemical staining for the skeletal muscle markers myogenin and MyoD1, collectively indicated a high suspicion of DICER1 syndrome. In this case, the cervical ERMS exhibited a soft and fragile mass, immunohistochemistry showed positive staining for myogenin and MyoD1, and gene sequencing showed mutations in Exon 25 c.5439G > C (a RNase IIIb hotspot regions). The diagnosis of DICER1 syndrome was definite. Due to its exophytic growth pattern, cervical ERMS can often be detected early in the early stages of the disease, which is associated with a relatively favorable prognosis [11, 12]. Devins et al. [13] analyzed 94 cervical ERMS patients, including nine with DICER syndrome, and all the patient had no extrauterine recurrence and none died of disease. However, in this case, the patient developed metastatic tumors in the pelvic retroperitoneum at the same time as the discovery of cervical neoplasm, experienced rapid recurrence and death despite standard therapy. This highlights a clinically aggressive subset. Based on molecular genetic analysis, we speculate that multiple gene mutations, may contribute to a more aggressive presentation and poorer prognosis in this instance. Literature reports indicate that a mutation in the DICER1 gene (c.5439G > C in E1813, a RNase IIIb hotspots) may suggest that these mutant cells can support migration, invasion and/or recolonization [14]. Additionally, dual mutations in DICER1 and TP53 or NF1 genes have been reported in DICER1‐related tumors [15, 16]. Generally, somatic alterations in NF1 or NF1 loss‐of‐function alterations are associated with the activation of the RAS/MAPK pathway, causing an uncontrolled cell growth and survival [17, 18]. Somatic TP53 mutations in ERMS have also been associated with poor prognosis and histologic anaplasia. Although DICER1 mutations may disrupt miRNA processing and promote tumor growth, NF1 and TP53 mutations further exacerbate this process by impairing tumor suppression. In this case, mutation in RNase IIIb hotspots and multiple gene mutations may be responsible for her initial retroperitoneal metastasis, as well as the subsequent pleural and mediastinal metastasis that occurred soon after chemotherapy. Overall, genetic counseling and disease monitoring for members of the DICER1 syndrome family are essential. This case report highlights a rare instance of DICER1 syndrome, ultimately diagnosed as DICER1 syndrome‐associated primary cervical ERMS. Based on the limited cases reported in the literature, no definitive conclusions have been drawn regarding prognosis.

We emphasize the importance of extended genetic testing to identify co‐existing mutations (e.g., in *TP53*, *NF-1*) that may influence tumor behavior and prognosis. We detail the necessity of instituting personalized, multidisciplinary monitoring plans, including regular clinical evaluations and targeted imaging of high‐risk organs, to enable early detection and intervention.

## Author Contributions

You‐bin Hu: project development and revision of the manuscript. Xiao‐qiang Wei: writing and revision of the manuscript. Qiu‐yang Wang: collection of clinical data. Tian Li: collection of clinical data.

## Funding

No funding was received for this manuscript.

## Disclosure

All authors read and approved the final manuscript before submission

## Ethics Statement

Written consent was obtained from the patient′s sister for the publication of their information and images. The study was approved by the Research Ethics Committee of Qingdao Women and Children′s Hospital, Women and Children′s Hospital of Qingdao University (QFELL‐YJ‐2023‐118).

## Conflicts of Interest

The authors declare no conflicts of interest.

## Supporting information


**Supporting Information** Additional supporting information can be found online in the Supporting Information section. Figure S1:Macroscopic appearance of cERMS in DICER1 syndrome: A polypoid mass protruding from the vagina (a), a hysteroscopy showed an exophytic and fragile mass with dendritic vessels (b), an exophytic mass (59 × 56 × 39 mm) occupying the entire vagina was located at the posterior wall of the cervical os as seen on the pelvic MRI (c), the MRI images demonstrated a well‐defined solid pelvic tumor (f), and a laparoscopic retroperitoneal tumor resection exhibited a lobulated mass without capsule (d and e).

## Data Availability

The data that support the findings of this study are available on request from the corresponding author. The data are not publicly available due to privacy or ethical restrictions.
