# Supplementary figures and images for: DICER1 Syndrome With Embryonal Rhabdomyosarcoma of the Uterine Cervix and Retroperitoneal Metastasis: A Case Report and Literature Review
Source: Case Rep Obstet Gynecol. 2026 Mar 11;2026:9718758. doi: 10.1155/crog/9718758 (PMC12978575; doi:10.1155/crog/9718758)

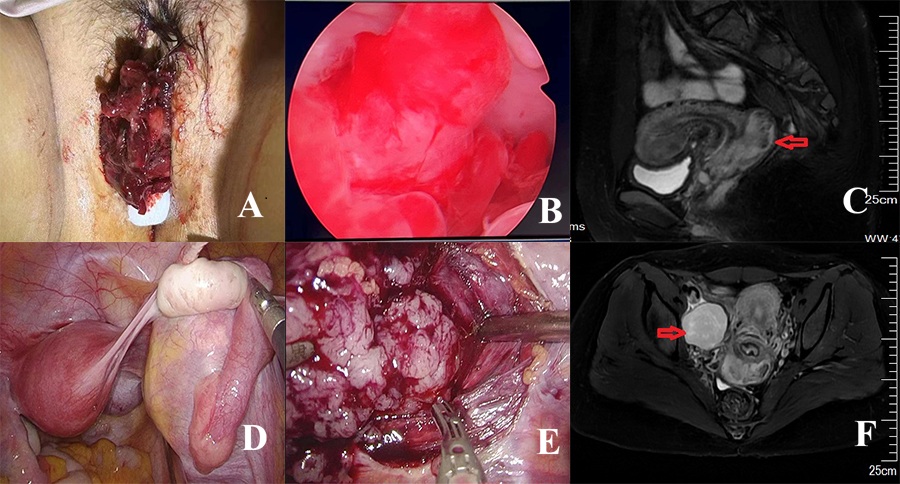

Supplement: Supplementary file 1 — Supporting Information Additional supporting information can be found online in the Supporting Information section. Figure S1:Macroscopic appearance of cERMS in DICER1 syndrome: A polypoid mass protruding from the vagina (a), a hysteroscopy showed an exophytic and fragile mass with dendritic vessels (b), an exophytic mass (59 × 56 × 39 mm) occupying the entire vagina was located at the posterior wall of the cervical os as seen on the pelvic MRI (c), the MRI images demonstrated a well‐defined solid pelvic tumor (f), and a laparoscopic retroperitoneal tumor resection exhibited a lobulated mass without capsule (d and e). [file CROG-2026-9718758-s001.jpg]
